# Role of Complement C5 in Experimental Blunt Chest Trauma-Induced Septic Acute Lung Injury (ALI)

**DOI:** 10.1371/journal.pone.0159417

**Published:** 2016-07-20

**Authors:** Miriam Kalbitz, Michael Karbach, Sonja Braumueller, Philipp Kellermann, Florian Gebhard, Markus Huber-Lang, Mario Perl

**Affiliations:** 1 Department of Traumatology, Hand-, Plastic-, and Reconstructive Surgery, Center of Surgery, University of Ulm, Ulm, Germany; 2 Orthopedic Trauma, BG-Trauma Center Murnau, Murnau, Germany; French National Centre for Scientific Research, FRANCE

## Abstract

**Background:**

Severe blunt chest trauma is associated with high mortality. Sepsis represents a serious risk factor for mortality in acute respiratory distress syndrome (ARDS). In septic patients with ARDS complement activation products were found to be elevated in the plasma. In single models like LPS or trauma complement has been studied to some degree, however in clinically highly relevant double hit models such as the one used here little data is available. Here, we hypothesized that absence of C5 is correlated with a decreased inflammatory response in trauma induced septic acute lung injury.

**Methods:**

12 hrs after DH in mice the local and systemic cytokines and chemokines were quantified by multiplex bead array or ELISA, activated caspase-3 by western blot. Data were analyzed using one-way ANOVA followed by post-hoc Sidak’s multiple comparison test (significance, p≤ 0.05).

**Results:**

In lung tissue interleukin (IL)-6, monocyte chemo attractant protein-1 (MCP-1) and granulocyte-colony stimulating factor (G-CSF) was elevated in both C5^-/-^ mice and wildtype littermates (wt), whereas caspase-3 was reduced in lungs after DH in C5^-/-^ mice. Systemically, reduced keratinocyte-derived chemokine (KC) levels were observed after DH in C5^-/-^ compared to wt mice. Locally, lung myeloperoxidase (MPO), protein, IL-6, MCP-1 and G-CSF in brochoalveolar lavage fluid (BALF) were elevated after DH in C5^-/-^ compared to wt.

**Conclusions:**

In the complex but clinically relevant DH model the local and systemic inflammatory immune response features both, C5-dependent and C5-independent characteristics. Activation of caspase-3 in lung tissue after DH was C5-dependent whereas local inflammation in lung tissue was C5-independent.

## Introduction

Severe chest trauma is associated with approximately one quarter of all trauma-related deaths [1]. The development of acute lung injury (ALI) in critically ill trauma patients independently contributes to elevated in-hospital mortality [2]. In addition, sepsis represents a serious risk factor for a mortality rate up to 50% in acute respiratory distress syndrome (ARDS) [3].

The complement system and its activation products play an important role in the clinical and experimental setting of sepsis [4, 5]. For attacking and lysing of gram-negative bacteria, the membrane attack complex (MAC, C5b-9) seems crucial, but also the anaphylatoxins C3a and C5a, which are major recruiter and activator of the first cellular line of defense. In experimental CLP-sepsis complement C5a receptor 1 knock out (C5aR1^-/-^) and C5a receptor 2 knock out (C5aR2^-/-^) mice had superior survival rates compared to wt [6]. Wt and C5 (C5^-/-^) knock out mice had comparable survival rates [7]. These observations suggest, that abrogated interaction of C5a with its receptors is somehow protective in polymicrobial sepsis but not the complete absence of C5 and its downstream complement products. Furthermore, C5^-/-^ mice were not able to form the terminal MAC, displayed in absence of hemolytic complement activity (CH-50) associated with reduced systemic bacterial clearance (400-fold) and tremendous bacterial load in C5^-/-^ mice in sepsis [7]. Whereas in rats blockade of C5a in sepsis induced by CLP was associated with reduced bacteremia and bacterial load in spleen and liver [8]. On the other hand elevated levels of the MAC were found in the plasma of septic patients that preceded the development of ARDS [9]. Clinical studies also revealed that complement activation occurs in lungs during ARDS development [10].

Blunt chest trauma induced PMN influx in the lungs [11–13]. Depletion of neutrophils in chest trauma-induced septic acute lung injury was beneficial and attenuated the degree of lung injury [12]. Neutrophil infiltration in the lung after blunt chest trauma has been shown to be C5a dependent [14]. After blunt chest trauma induced bilateral lung injury local and systemic activation of the complement system has been observed [14, 15]. Systemic activation of the complement system with generation of the anaphylatoxin C5a results in activation of neutrophils (PMN) with sequestration and adhesion to the pulmonary capillary endothelium. This may lead to damage of the vascular endothelial cells and thereby contribute to the development of ALI [16]. In the model of IgG immune complex-induced ALI C5aR was up-regulated on lung epithelial cells and adding significantly to the development of inflammation and lung injury [17]. In contrast lipopolysaccharides (LPS)-induced ALI fully developed in C5^-/-^ mice during sepsis compared to complement sufficient animals [18], suggesting that LPS-induced ALI is rather C5 independent. On the other hand in a model of pulmonary contusion followed by a second hit of LPS instillation, inhibition of C5a/C5aR correlated with a decreased inflammatory response [15].

However, the current studies provide novel evidence that the activation of caspase-3 in lung tissue after DH is C5-dependent whereas local inflammation in lung tissue is C5-independent.

## Materials and Methods

### Animals

All investigative procedures and the animal facilities conformed by the guide of care and use of laboratory animals published by the US National Institutes of Health. The study protocol was approved by the federal authorities for animal research, Tübingen, Germany (Permit No. 958). Adult, male, C57BL/6 (wt, C5^+/+^) and C5 deficient (C5^-/-^) C57BL/6 mice (8–9 weeks), 25 ± 3 g, were obtained from Jackson Laboratories (Bar Harbor, ME). Animals were allowed free access to water and food before and after chest trauma and sepsis.

### Animal preparation

Mice were anesthetized with 2.5% sevoflurane (Abott, Wiesbaden, Germany) and 97.5% oxygen mixture under continuous flow of 0.5 l/min. Buprenorphine (Essex, Pharma, Munich, Germany) was administered subcutaneously (0.03 mg/kg body weight) immediately after trauma, sepsis or sham procedure and every 8 hrs thereafter. Animals were allowed free access to water and food before and after experimental procedures. The animals were observed frequently every 12 hours. During the observation period the animals were evaluated on the basis of following criteria for euthanasia: weight loss >20% compared to baseline weight, hypothermia, cyanosis, shivering, seizures, lost of motion and paralysis. At the end of the observation period the anesthetized mice (2,5% sevoflurane and 97.5% oxygen mixture under continuous flow of 0.5 l/min) were euthanized by exsanguination via cardiac puncture. During the study no animal underwent euthanasia prior the experimental endpoint. Three animals died directly after blunt chest trauma. In our cohort so far deaths are due to lethal chest injury such as pericardial effusion.

### Blunt chest trauma

Bilateral lung contusion was induced by a single blast injury in anaesthetized mice as described previously [19, 20]. In brief, opening of a high-speed valve (Hee-D-24, Festo, Esslingen, Germany) delivered compressed air into the upper section of a cylinder. The upper compartment is separated to the lower section with a Mylar polyester film (Du Pont de Nemur, Bad Homburg, Germany). The polyester membrane ruptured at standardized pressure releasing a defined blast wave in the lower compartment of the cylinder, centered on the ventral thorax of the animal. The level of pulmonary contusion was chosen based on histologic, cardiopulmonary and immunologic changes in earlier studies, sufficient to induce a profound local and systemic inflammatory response, but without being lethal itself [19].

### Cecal ligation and puncture (CLP) -sepsis

Polymicrobial mid grade sepsis or sham procedure was induced 24 hrs after blunt chest trauma. CLP was performed as described previously [21]. In anesthetized mice mid line laparotomy was performed. The cecum was ligated 10 mm from its apex and double punctured with a 22-gauge needle. A minimal amount of bowel content was extruded and the cecum was relocated. The abdominal incision was closed in layers using 4–0 sutures (Ethilon, Ethicon GmbH, Norderstedt, Germany). Mice received a subcutaneous injection of Ringer’s lactate (40 ml/kg body weight) immediately after surgery. Sham animals were treated similarly without ligation or puncture of the cecum.

### Experimental groups and design

Wt and C5^-/-^ mice were randomized into two groups (n = 7). One group was subjected to blunt chest trauma. The other group of animals served as sham controls. 24 hrs after blunt chest trauma CLP or sham procedure were induced respectively.

### Bronchoalveolar lavage fluid (BALF) and lung tissue

At the end of the study (36 hrs after blunt chest trauma and 12 hrs after CLP) animals were sacrificed, trachea of the animals was carefully exposed and cannulated using 0.58 mm x 0.2 mm internal diameter polyethylene tubing (Merck, Darmstadt, Germany). Then, 0.5 ml ice cold PBS (Gibco) was carefully injected and recovered three times and stored on ice after addition of 1μl of proteinase inhibitor cocktail (Sigma, Taufkirchen, Germany) per 100 μl of BALF. BALF was centrifuged at 500 x g for 10 min at 4°C. Supernatant fluid was stored at -80°C until analysis. Preparation of lung homogenates for cytokine measurements was performed as described previously [11, 22]. In brief, lungs were gently flushed through the vascular system via injection of 2mL of ice-cold PBS (Gibco, Eggenstein, Germany) into right ventricle, than harvested, immediately frozen in liquid nitrogen and stored at -80°C until analysis. Lung tissues were weighted, transferred to different tubes on ice containing protease inhibitor cocktail (Sigma). The lung tissues were homogenized (ULTRA-TURRAX, IKA, Staufen, Germany), sonicated (UW2070, Bandelin, Berlin, Germany) on ice and centrifuged at 16,000G for 15 min at 4°C. Protein concentrations were determined using BCA Protein Assay Kit (Pierce, Rockford, IL).

### Plasma preparation

Blood obtained by cardiac puncture was placed in micro centrifuge tubes containing heparin and immediately spun at 3,000 x g at 4°C for 10 min. Heparin-plasma was stored at -80°C until analysis.

### Cytokine and chemokine analysis

BALF, plasma and lung homogenate concentrations of IL-6 and G-CSF were analyzed by cytometric bead array (Bio-Plex Pro Assay, Bio-Rad Laboratories GmbH, Munic, Germany). MCP-1 (BD OptEIA^™^, Becton Dickinson & Company, Franklin Lakes, USA), and KC (DuoSet^®^ ELISA Kit, R&D Systems, Minneapolis, USA) concentrations were measured by sandwich-enzyme-linked immunosorbent assay technique (ELISA). Levels below the detection limit of ELISA or cytometric bead array were set to zero for statistical purposes. The cytometric bead array utilized has been validated for BALF, plasma and tissue lysates. The KC and MCP1 ELISA kits were used in earlier studies to determine concentration in lung tissue homogenates [11].

### Lung active caspase-3 Western blotting

Active caspase-3 was quantified in lung tissue via Western blotting as described previously [22]. Briefly, membranes were incubated with polyclonal rabbit anti-mouse cleaved caspase-3 (Asp175) antibody (Cell Signaling, Danvers, Mass) diluted 1:500 at 4°C over night, followed by incubation with anti-rabbit IgG, horseradish peroxidase (HRP)-linked secondary antibody (Cell Singaling) diluted 1:1,500 for 1h and developed using enhanced chemiluminescence technique (ChemiGlow; Alpha Innotech, San Leandro, CA). To ensure equal amounts of protein loading, the blots were stripped (Strong Antibody Stripping Solution, Chemicon, Temecula, CA) and re-probed with β- actin antibody (Cell Signaling) diluted 1:1000, followed by incubation with anti-rabbit IgG, HRP-linked secondary antibody (Cell Signaling) diluted 1:1,500 for 1hr and developed using enhanced chemiluminescence technique (ChemiGlow). For densitometry analysis Image J was used [23].

### BALF protein concentration

Protein concentrations in BALF were determined using BCA Protein Assay Kit (Pierce, Rockford, IL) as recommended by the manufacturer with a microplate reader (Tecan GmbH, Gröding, Austria).

### Lung Myeloperoxidase (MPO)

Lung MPO activity was quantified as described previously [24]. In brief, lung tissue was homogenized (Ultraturrax T25, Jahnke und Kunkel, Staufen, Germany) in 1.5 ml buffer containing 10.35 g KH_2_PO_4_ (Merck) in 950 mL distilled water adjusted to pH 5.4 using 0.91 g K_2_HPO_4_ (Merck) in 50 ml distilled water and 0.5% hexadecyltriethylammonium bromide (Sigma). Tissue homogenates were incubated at 60°C for 2 hrs followed by centrifugation at 3,950 x g at room temperature for 15 min. 25 μl tissue or standard solution (Novabiochem, Schwalbach, Germany) was mixed with 25 μl tetramethylbenzidine (Sigma) and 200 μl of 0.002% H_2_O_2_ (Fluka, Deisenhofen, Germany) and incubated at 37°C for 5 min. Extinction values were read at 450 nm and MPO activity was calculated on the basis of standard curves. Thereafter perchloric acid precipitation was performed and protein concentration was determined using BCA Protein Assay Kit as recommended by the manufacturer.

### Reagents and chemicals

All materials were obtained from Sigma Chemical Co. (St. Louis, MO) unless otherwise indicated.

### Statistical analysis

All values were expressed as mean ± SEM. Data were analyzed using one-way ANOVA followed by Sidak’s multiple comparison test. Results were considered statistically significant where p≤ 0.05.

## Results

### Double hit (DH)-induced local tissue inflammation in lungs

**Fig 1:**
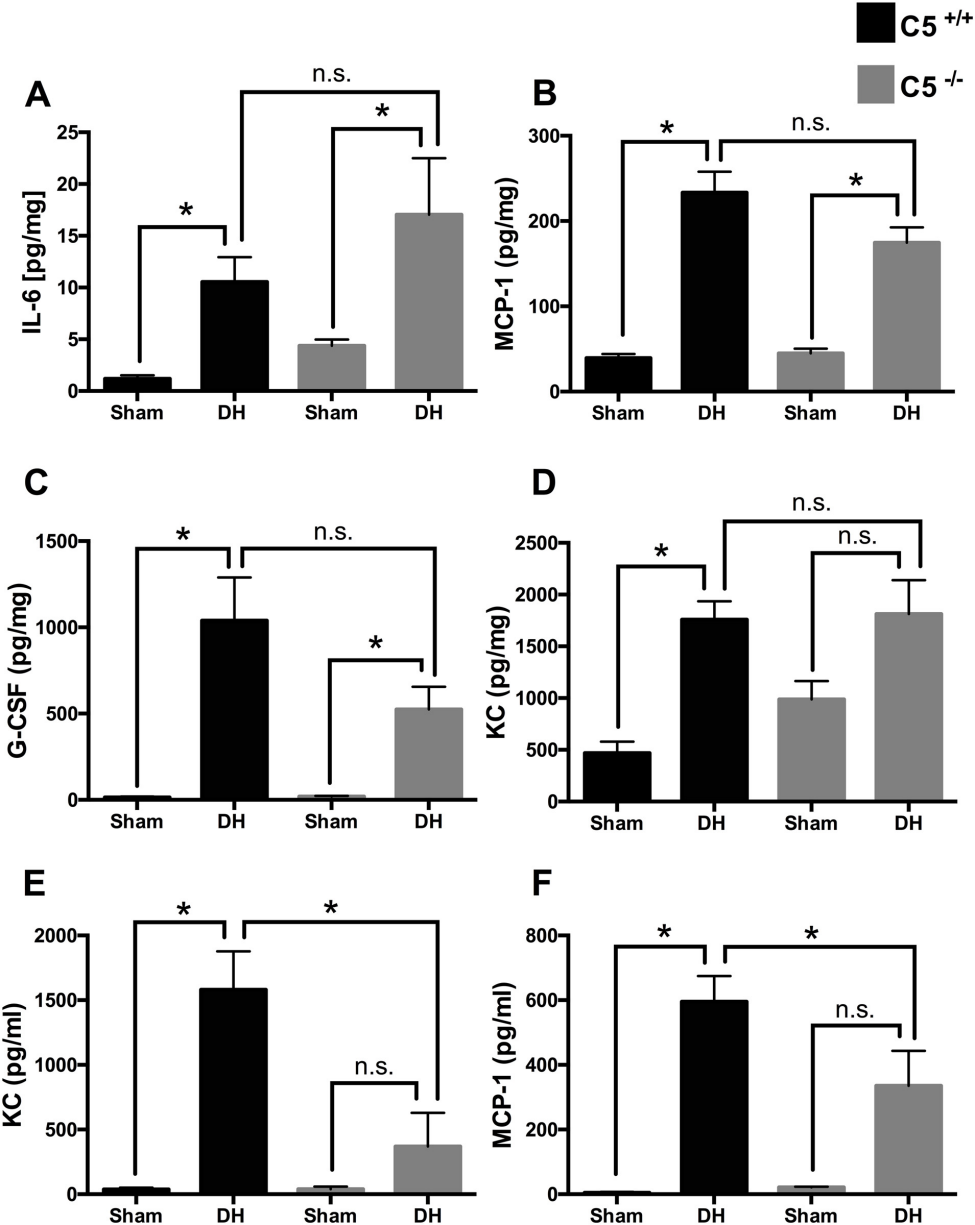
Cytokines and chemokines in lung tissue after sham procedure or double hit (DH) in wild type (wt) mice (black bars) or in absence of C5 (C5^-/-^) mice (grey bars). Systemic effects of the absence of complement C5 in double hit (DH). **A**. Increased IL-6 levels in lung tissue of wt and in C5^-/-^ mice after DH compared to sham. **B**. Increased MCP-1 levels in lung tissue of wt and in C5^-/-^ after DH compared to sham. **C**. Increased G-CSF levels in lung tissue of wt and in C5^-/-^ mice after DH compared to sham. **D**. Increased KC levels in lung tissue of wt mice. **E**. Increased KC plasma concentration in wt mice after DH; decreased KC plasma concentrations after DH in C5^-/-^ compared wt mice. **F**. Increased MCP-1 plasma concentrations in wt mice after DH, decrease of MCP-1 after DH in absence of C5. For each bar, n = 7 separate mice. * p<0.05, n.s. = not significant.

Levels of pro-inflammatory mediators were quantitated in lung tissue homogenates after blunt chest trauma followed by sepsis (Fig 1). In animals after DH IL-6 (frame A), MCP-1 (frame B), G-CSF (frame C) and KC (frame D) were increased compared to sham procedure in wt mice. IL-6, MCP-1 and G-CSF were likewise increased after DH in C5^-/-^ mice. KC was increased after DH in wt (frame D). No differences in lung tissue concentrations were observed in IL-1β and regulated upon activation normal T cell expressed and presumably secreted (RANTES) after DH (data not shown). However, there was no statistically significant difference in the measured cytokines and chemokines (frame A-D) in the absence or presence of C5.

### Amelioration of the systemic inflammatory response after DH in C5^-/-^ mice

As expected, at 12 hrs after CLP and 36 hrs after blunt chest trauma respectively, plasma KC (Fig 1E) and MCP-1 (Fig 1F) were increased in wt. In contrast, in C5^-/-^ mice the KC and MCP-1 increase was significantly attenuated after DH.

### Reduced caspase-3 in lung tissue of C5 deficient mice after DH

**Fig 2:**
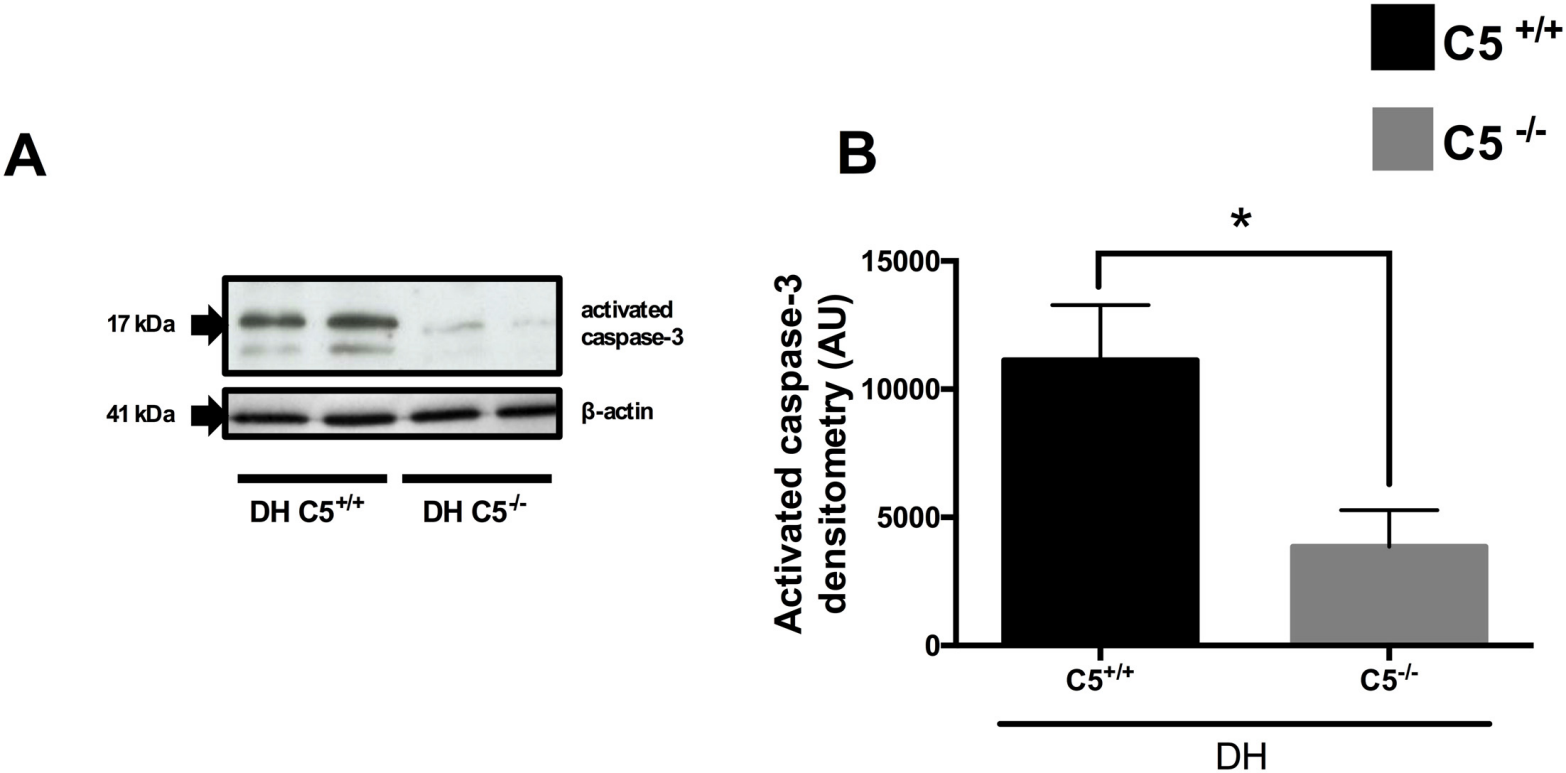
Activated caspase-3 in lung tissue in C5^+/+^, wt (black bar) or C5^-/-^ (grey bar) mice after sham procedure or double hit (DH). **A**. Representative western blot of activated caspase-3 in two wt and two C5^-/-^ mice after DH and corresponding western blot of anti-β-actin. **B**. Densitometry of three independent western blots for activated caspase-3. For each bar, n = 7 separate mice. * p<0.05.

In regard to assess apoptotic events activated caspase-3 as a key pro-apoptotic indicator was determined in lung tissue. In C5^-/-^ mice cleaved/active caspase-3 was significantly reduced after double hit compared to wt mice as determined by Western Blot analysis. Blots were stripped and re-probed with anti-β-actin antibody (Fig 2A and 2B).

### Partial C5 dependency of the local cytokine and chemokine profile

**Fig 3:**
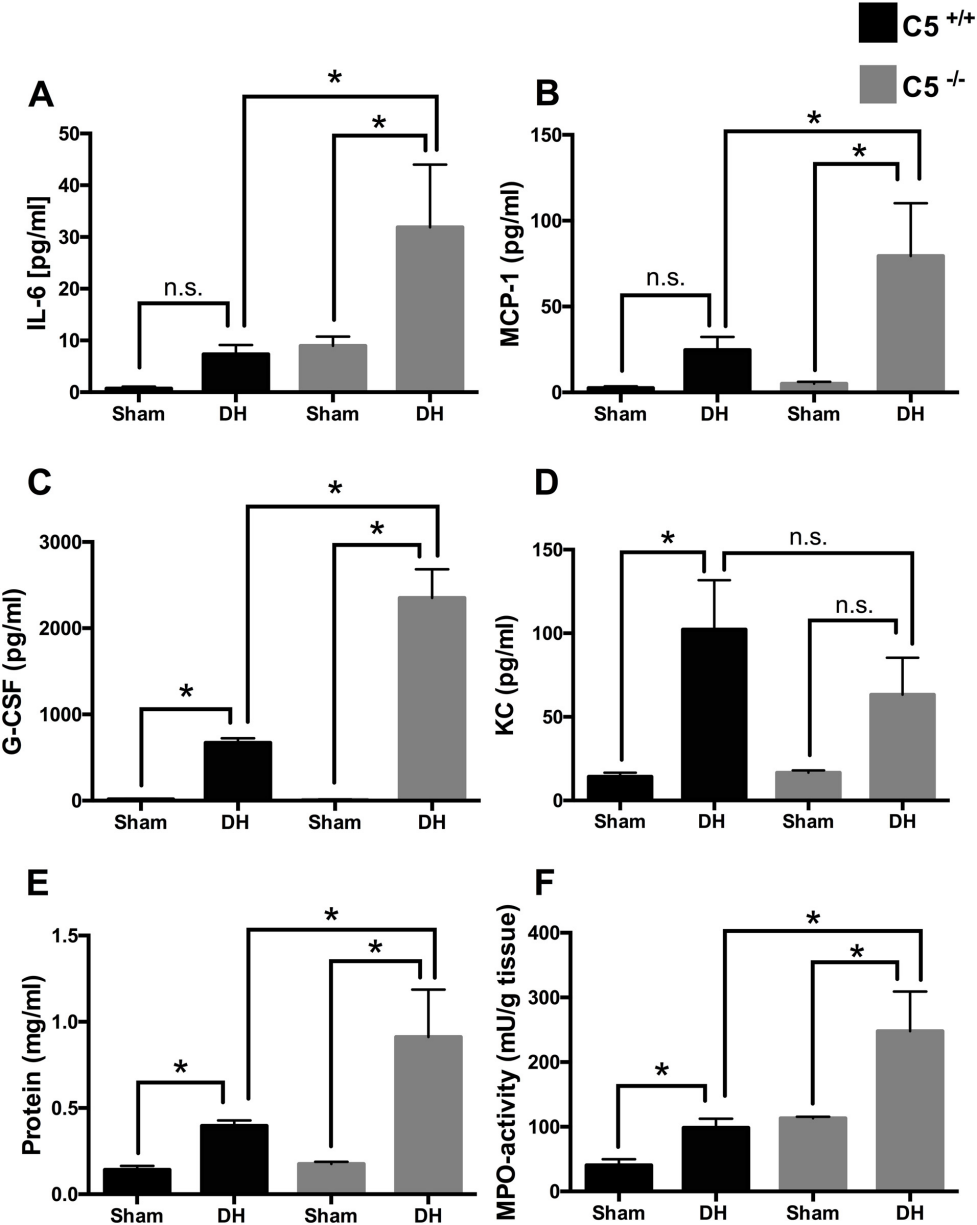
Bronchoalveolar lavage fluid (BALF) cytokine, chemokine and protein concentrations in mice after sham procedure or double hit (DH) in C5^+/+^ (wt) mice (black bars) and C5^-/-^ mice (grey bars) and MPO activity in lung tissue. Increased IL-6 BALF concentration in C5^-/-^ mice after DH compared to sham and compared to DH in wt mice. **B**. Increased MCP-1 levels after DH in absence of C5 compared to sham and compared to DH in wt mice. **C**. Increased G-CSF BALF concentration after DH in presence and absence of C5. Further increase of G-CSF concentration after DH in absence of C5 compared to wt. **D**. Increased KC levels in wt mice after DH. **E**. Increased protein concentrations in bronchoalveolar lavage fluids (BALF) after DH in both wt (black bars) and in absence of C5 (grey bars). Further increase in BALF protein concentrations in C5^-/-^ after DH compared to DH in wt mice. **F**. MPO activity in lung tissue is increased after DH compared to sham in both wt and in absence of C5 and further increased after DH in absence of C5. For each bar, n = 7 separate mice. * p<0.05, n.s. = not significant.

The local generation of proinflammatory mediators was quantitated in BALF after blunt chest trauma followed by sepsis (Fig 3). In animals after DH (i.e. 36 hrs after blunt chest trauma and 12 hrs after CLP, respectively) IL-6 (frame A) and MCP-1 (frame B) were increased in C5^-/-^ mice compared to sham procedure and especially compared to DH in C5^+/+^ animals. G-CSF (frame C) was increased after DH in wt and C5^-/-^ mice compared to mice after sham procedure. G-CSF after DH in C5^-/-^ was further increased compared to wt mice. KC was significantly increased in wt (frame D). IL1-β and RANTES protein were not significantly increased in both wt and C5^-/-^ (data not shown).

### Increase of the pulmonary vascular leakage and MPO activity in the absence of C5

Pulmonary vascular leakage as assessed by total BALF protein after blunt chest trauma and sepsis was significantly increased in wt and further aggravated in C5^-/-^ mice (Fig 3, frame E). PMN activation as assessed by MPO activity in lung tissue (Fig 3, frame F) 36 hours after blunt chest trauma and 12 hours after induction of sepsis, was increased after DH in wt and C5^-/-^ mice.

## Discussion

In the present study the role of C5 as a main component of the complement system was determined in a DH model of ALI induced by blunt chest trauma and subsequent polymicrobial sepsis.

Blunt chest trauma was shown to cause severe immune dysfunction of splenocytes, macrophages and monocytes associated with increased susceptibility to infectious complications and poor survival after pulmonary contusion when followed by a septic insult [25]. Reflecting the clinical scenario of ALI encountered in severely injured patients, presence of sepsis represents an overwhelming risk factor for mortality [3]. In contrast to DH models of pulmonary contusion and i.t. LPS administration as a second hit [15] the present DH model employs CLP-induced polymicrobial sepsis, which has been described as “gold standard” to study sepsis in rodents [26]. It is important to note that the two models are rather different. Main difference of the present study to recently published studies, using a DH model of pulmonary contusion and i.t. administration of LPS, is the local second hit with LPS from a single E. coli strain [15] vs. the systemic inflammatory response based on CLP-induced polymicrobial sepsis. In CLP-induced polymicrobial sepsis manifold pathogen associated molecular patterns (PAMPs) are present systemically due to leakage of peritoneal microbial flora into the peritoneum. Furthermore, danger associated molecular patterns (DAMPs) due to tissue trauma by laparotomy and necrosis caused by ligation of the cecum are present. Together this is associated with a different inflammation profile and time course after CLP compared to LPS as a second insult [27]. Systemic LPS injection results in a strong and immediate increase of several pro-inflammatory cytokines such as IL-6, TNF and IL-1β compared to a prolonged cytokine increase after CLP which resembles the progression and characteristics of human sepsis [26]. These systemic mediators further stimulate the immune system, previously significantly primed by blunt chest trauma [25]. In experimental DH survival in wild type mice was 50%, here fatalities occurred within the first 7 days. Whereas animals subjected to chest trauma alone survival rate was nearly 90% and in wild type mice midgrade sepsis alone resulted in around 75% survival. Indicating that the combined challenge of pulmonary contusion and moderate sepsis significantly decreased survival compared to blunt chest trauma or sepsis alone [25].

In lung tissue cytokine and chemokine concentrations were elevated 36 hrs after blunt chest trauma and 12 hrs after induction of sepsis by CLP in both presence and absence of C5 (Fig 1) suggesting that CLP-sepsis acts as a relevant systemic second hit to maintain and reinforce inflammation in the lungs. Simultaneously in BALF IL-6, MCP-1 and G-CSF were amplified in absence of C5 and its activation product C5a (Fig 3). In accordance C5aR2-deficient mice released significantly more IL6 and TNF than wt animals following immune complex injury of the lungs [28]. These results could be due to missing interaction of C5a with its C5aR2 receptor in absence of C5 and consecutively loss of anti-inflammatory effect. Increased G-CSF in BALF after DH was increased and further enhanced in C5^-/-^ mice, which therefore might be due to the loss of local anti-inflammatory effects. These results closely reflect the human situation after lung contusion with inhomogeneous tissue damage/bleeding patterns within the different compartments of the lung. Cell type composition and number recruited to the alveolar space differ from the cell variety in the lung tissue, which might explain the different cytokine profile.

Reduced cytokine and chemokine concentrations in plasma of C5^-/-^ mice in present DH model of blunt chest trauma and polymicrobial sepsis (Fig 1) are in accordance with earlier studies which showed diminished plasma levels of pro-inflammatory mediators such as Il-6 and MCP-1 and anti-inflammatory mediators such as IL-1 receptor antagonist (IL-1Ra) and IL-10 in C5^-/-^ mice 24 hrs after intermediate-grade CLP-induced sepsis associated with greatly impaired bacterial clearance [7]. Mechanistically, C5a may function as suppressor of IL-6 gene expression in the endothelium, as shown in human umbilical vein endothelial cells [29]. In sepsis blockade of either C5aR 1, C5aR2 or C5a were associated with decreased serum levels of IL-6 [4, 30]. In the present DH setting plasma levels of G-CSF in wt mice were elevated compared to sham operated wt mice and attenuated in C5 deficient mice objected to DH. This finding is in accordance to a previous report, which showed substantially lower plasma levels of G-CSF during sepsis in C5aR1- and C5aR2-deficient as compared to wt mice [31]. C5a promoted production of IL-10 from LPS-activated PMNs in which G-CSF release was independent of inhibitory effects of IL-10 [31]. In rats infusion of human recombinant C5a caused transient neutropenia [32]. Reciprocal effects of C5a, as an inducer of pro-inflammatory cytokines/chemokines have been reported in other cell types such as alveolar epithelial cells [33] and microvascular endothelial cells [34].

In patients with ARDS enhanced levels of pro-inflammatory cytokines and PMNs were detected in BALF [35]. In the lungs KC chiefly derived from macrophages acting as neutrophil chemo-attractant and playing important pathophysiological roles in ALI [36]. In accordance to present DH model, KC was found to be elevated in lungs and plasma in a mouse model of hemorrhage priming for ALI [37]. In C5^-/-^ mice KC in plasma was significantly reduced after DH compared to wt mice. These findings harmonize with earlier CLP-studies where the increased levels of KC mRNA expression were almost abolished after application of anti-C5a antibody [38]. In the present study reduced concentrations of KC in combination with increased MPO activity in absence of C5 was observed, suggesting that other chemo-attracting mechanisms (such as IL-6) were involved in neutrophil recruitment into the alveolar space after DH.

Besides inflammation, apoptosis has been shown to contribute to the development of chest trauma-induced septic ALI [13]. In BALF of patients with ALI increased concentrations of Fas and Fas ligand were observed [39]. Increased lung tissue apoptosis was observed in a similar DH model of blunt chest trauma followed by an earlier CLP [12]. In present study in C5^-/-^ mice activated caspase-3 in lung tissue was reduced compared to C5^+/+^ mice after DH (Fig 2). These results were in accordance to previous studies where apoptosis after CLP was markedly reduced after C5a blockade in other cell types such as thymocytes [40]. Furthermore, C5a blockade by anti-C5a antibody markedly restored the susceptibility of neutrophils to undergo apoptosis after CLP [41]. This could in turn be one possible explanation for increased lung MPO activity in C5^-/-^ mice after DH in the present study.

Limitations of the present study are the rare time points. Furthermore, in the DH setting no comparison between C5aR1^-/-^ and C5aR2^-/-^ mice was performed, which *per se* exhibit better survival rates after CLP compared to wt and C5^-/-^ mice in earlier studies. MPO does not accurately reflect the number of PMNs but rather the activation status. So we can only speculate that PMNs in absence of C5a in C5 knock out mice are attracted by another not yet defined chemotactic mechanism. Furthermore, C5^-/-^ mice reveled severe neutropenia following CLP compared to wt mice, which showed significant increase of blood neutrophils [7]. To further access the pro- and anti-inflammatory profile of multiple mediators in regard to C5-dependency, a gene array or proteomic approach could define more proteins involved in a hypothesis-free driven manner.

In present DH model consisting of blunt chest trauma and polymicrobial sepsis, a spatial modulation of the inflammatory replay appeared in both a C5-dependent (e.g. activation of caspase-3, plasma and BALF cytokines and chemokines) and C5-independent manner (e.g. several lung tissue cytokines and chemokines). The results are indicative of a complex innate immune response in this model simulating the clinical setting of polymicrobial septic ALI including other branches of the innate immune system such as toll-like receptors (TLRs) [42, 43]. In conclusion, the data suggest in the DH model that absence of C5 may intensify mediator production in BALF as well as increased evidence of acute lung injury. This might be due to the abolition of suppressed mediator release from PMNs and macrophages in the presence of high C5a levels [31]. However, additional studies are needed to further clarify underlying pathomechanisms.

## Abbreviations

ALI: acute lung injury
ARDS: acute respiratory distress syndrom
BALF: bronchoalveolar lavage fluid
C5a: complement component 5a
C5aR: complement component 5a receptor 1
C5L2: complement component 5a receptor 2
CH-50: hemolytic complement activity
CLP: cecal ligation and puncture
DAMPs: danger associated molecular patterns
DH: double hit
G-CSF: granulocyte-colony stimulating factor
HRP: horseradish peroxidase
IL: interleukin
KC: keratinocyte-derived chemokine
LPS: lipopolysaccharide
MAC: membrane attack complex
MCP-1: monocyte chemo attractant protein-1
MPO: myeloperoxidase
PAMPs: pathogen associated molecular patterns
PEM: peritoneal elicited macrophages
PMN: polymorphonuclear leukocyte
RANTES: regulated upon activation normal T cell expressed and presumably secreted
wt: wild type

## References

[1] Stellin G. Survival in Trauma Victims with Pulmonary Contusion. Am Surgeon. 1991;57(12):780–4. WOS:A1991GU98400009. 1746794

[2] Shah CV, Localio AR, Lanken PN, Kahn JM, Bellamy S, Gallop R, et al The impact of development of acute lung injury on hospital mortality in critically ill trauma patients. Critical Care Medicine. 2008;36(8):2309–15. WOS:000258271100013. 18664786 10.1097/CCM.0b013e318180dc74

[3] Brun-Buisson C, Minelli C, Bertolini G, Brazzi L, Pimentel J, Lewandowski K, et al Epidemiology and outcome of acute lung injury in European intensive care units. Results from the ALIVE study. Intensive Care Med. 2004;30(1):51–61. 10.1007/s00134-003-2022-6 . 14569423

[4] Riedemann NC, Guo RF, Neff TA, Laudes IJ, Keller KA, Sarma VJ, et al Increased C5a receptor expression in sepsis. J Clin Invest. 2002;110(1):101–8. Epub 2002/07/03. 10.1172/JCI15409 12093893 PMC151030

[5] Riedemann NC, Guo RF, Bernacki KD, Reuben JS, Laudes IJ, Neff TA, et al Regulation by C5a of neutrophil activation during sepsis. Immunity. 2003;19(2):193–202. Epub 2003/08/23. S1074761303002061 [pii]. . 12932353 10.1016/s1074-7613(03)00206-1

[6] Rittirsch D, Flierl MA, Nadeau BA, Day DE, Huber-Lang M, Mackay CR, et al Functional roles for C5a receptors in sepsis. Nat Med. 2008;14(5):551–7. Epub 2008/05/06. nm1753 [pii] 10.1038/nm1753 18454156 PMC2753858

[7] Flierl MA, Rittirsch D, Nadeau BA, Day DE, Zetoune FS, Sarma JV, et al Functions of the complement components C3 and C5 during sepsis. Faseb J. 2008;22(10):3483–90. Epub 2008/07/01. fj.08-110595 [pii] 10.1096/fj.08-110595 18587006 PMC2537435

[8] Czermak BJ, Sarma V, Pierson CL, Warner RL, Huber-Lang M, Bless NM, et al Protective effects of C5a blockade in sepsis. Nat Med. 1999;5(7):788–92. Epub 1999/07/08. 10.1038/10512 . 10395324

[9] Langlois PF, Gawryl MS. Accentuated Formation of the Terminal C5b-9 Complement Complex in Patient Plasma Precedes Development of the Adult Respiratory-Distress Syndrome. American Review of Respiratory Disease. 1988;138(2):368–75. WOS:A1988P626400023. 3264125 10.1164/ajrccm/138.2.368

[10] Robbins RA, Russ WD, Rasmussen JK, Clayton MM. Activation of the Complement-System in the Adult Respiratory-Distress Syndrome. American Review of Respiratory Disease. 1987;135(3):651–8. WOS:A1987G356300027. 3826891 10.1164/arrd.1987.135.3.651

[11] Perl M, Chung CS, Lomas-Neira J, Rachel TM, Biffl WL, Cioffi WG, et al Silencing of Fas, but not caspase-8, in lung epithelial cells ameliorates pulmonary apoptosis, inflammation, and neutrophil influx after hemorrhagic shock and sepsis. Am J Pathol. 2005;167(6):1545–59. 10.1016/S0002-9440(10)61240-0 16314469 PMC1613198

[12] Perl M, Hohmann C, Denk S, Kellermann P, Lu DP, Braumuller S, et al Role of Activated Neutrophils in Chest Trauma-Induced Septic Acute Lung Injury. Shock. 2012;38(1):98–106. WOS:000305501100016. 22552016 10.1097/SHK.0b013e318254be6a

[13] Weckbach S, Hohmann C, Denk S, Kellermann P, Huber-Lang MS, Baumann B, et al Apoptotic and inflammatory signaling via Fas and tumor necrosis factor receptor I contribute to the development of chest trauma-induced septic acute lung injury. Journal of Trauma and Acute Care Surgery. 2013;74(3):792–800. WOS:000316321900019. 23425737 10.1097/TA.0b013e31827a3655

[14] Flierl MA, Perl M, Rittirsch D, Bartl C, Schreiber H, Fleig V, et al The role of C5a in the innate immune response after experimental blunt chest trauma. Shock. 2008;29(1):25–31. Epub 2007/07/11. . 17621257 10.1097/shk.0b013e3180556a0b

[15] Hoth JJ, Wells JD, Jones SE, Yoza BK, McCall CE. Complement mediates a primed inflammatory response after traumatic lung injury. J Trauma Acute Care Surg. 2014;76(3):601–8; discussion 8–9. . 24553525 10.1097/TA.0000000000000129 PMC4426490

[16] Bosmann M, Ward PA. Role of C3, C5 and anaphylatoxin receptors in acute lung injury and in sepsis. Adv Exp Med Biol. 2012;946:147–59. Epub 2011/09/29. 10.1007/978-1-4614-0106-3_9 . 21948367 PMC3372066

[17] Sun L, Guo RF, Gao H, Sarma JV, Zetoune FS, Ward PA. Attenuation of IgG immune complex-induced acute lung injury by silencing C5aR in lung epithelial cells. Faseb J. 2009;23(11):3808–18. Epub 2009/07/22. fj.09-133694 [pii] 10.1096/fj.09-133694 19620403 PMC2775006

[18] Rittirsch D, Flierl MA, Day DE, Nadeau BA, McGuire SR, Hoesel LM, et al Acute lung injury induced by lipopolysaccharide is independent of complement activation. Journal of Immunology. 2008;180(11):7664–72. WOS:000257507300066. 10.4049/jimmunol.180.11.7664 PMC2753408 18490769

[19] Knoferl MW, Liener UC, Seitz DH, Perl M, Bruckner UB, Kinzl L, et al Cardiopulmonary, histological, and inflammatory alterations after lung contusion in a novel mouse model of blunt chest trauma. Shock. 2003;19(6):519–25. ISI:000183006900005. 12785006 10.1097/01.shk.0000070739.34700.f6

[20] Knoferl MW, Liener UC, Perl M, Bruckner UB, Kinzl L, Gebhard F. Blunt chest trauma induces delayed splenic immunosuppression. Shock. 2004;22(1):51–6. . 15201702 10.1097/01.shk.0000127684.64611.5c

[21] Rittirsch D, Huber-Lang MS, Flierl MA, Ward PA. Immunodesign of experimental sepsis by cecal ligation and puncture. Nat Protoc. 2009;4(1):31–6. Epub 2009/01/10. nprot.2008.214 [pii] 10.1038/nprot.2008.214 19131954 PMC2754226

[22] Perl M, Chung CS, Perl U, Lomas-Neira J, de Paelpe M, Cioffi WG, et al Fas-induced pulmonary apoptosis and inflammation during indirect acute lung injury. Am J Resp Crit Care. 2007;176(6):591–601. 10.1164/rccm.200611-1743OC ISI:000249646100009. PMC1994224 17600273

[23] Schneider CA, Rasband WS, Eliceiri KW. NIH Image to ImageJ: 25 years of image analysis. Nature methods. 2012;9(7):671–5. Epub 2012/08/30. . 22930834 10.1038/nmeth.2089 PMC5554542

[24] Weckbach S, Hohmann C, Braumueller S, Denk S, Klohs B, Stahel PF, et al Inflammatory and apoptotic alterations in serum and injured tissue after experimental polytrauma in mice: Distinct early response compared with single trauma or "double-hit" injury. Journal of Trauma and Acute Care Surgery. 2013;74(2):489–98. ISI:000316321200037. 23354243 10.1097/TA.0b013e31827d5f1b

[25] Perl M, Gebhard F, Bruckner UB, Ayala A, Braumuller S, Buttner C, et al Pulmonary contusion causes impairment of macrophage and lymphocyte immune functions and increases mortality associated with a subsequent septic challenge. Crit Care Med. 2005;33(6):1351–8. . 15942355 10.1097/01.ccm.0000166352.28018.a9

[26] Dejager L, Pinheiro I, Dejonckheere E, Libert C. Cecal ligation and puncture: the gold standard model for polymicrobial sepsis? Trends Microbiol. 2011;19(4):198–208. 10.1016/j.tim.2011.01.001 . 21296575

[27] Remick DG, Newcomb DE, Bolgos GL, Call DR. Comparison of the mortality and inflammatory response of two models of sepsis: lipopolysaccharide vs. cecal ligation and puncture. Shock. 2000;13(2):110–6. . 10670840 10.1097/00024382-200013020-00004

[28] Gerard NP, Lu B, Liu P, Craig S, Fujiwara Y, Okinaga S, et al An anti-inflammatory function for the complement anaphylatoxin C5a-binding protein, C5L2. The Journal of biological chemistry. 2005;280(48):39677–80. Epub 2005/10/06. 10.1074/jbc.C500287200 . 16204243

[29] Monsinjon T, Gasque P, Chan P, Ischenko A, Brady JJ, Fontaine MC. Regulation by complement C3a and C5a anaphylatoxins of cytokine production in human umbilical vein endothelial cells. Faseb J. 2003;17(9):1003–14. 10.1096/fj.02-0737com . 12773483

[30] Hopken U, Mohr M, Struber A, Montz H, Burchardi H, Gotze O, et al Inhibition of interleukin-6 synthesis in an animal model of septic shock by anti-C5a monoclonal antibodies. Eur J Immunol. 1996;26(5):1103–9. 10.1002/eji.1830260522 . 8647174

[31] Bosmann M, Haggadone MD, Zetoune FS, Sarma JV, Ward PA. The interaction between C5a and both C5aR and C5L2 receptors is required for production of G-CSF during acute inflammation. European Journal of Immunology. 2013;43(7):1907–13. 10.1002/Eji.201243075 WOS:000327695500025. 23575697 PMC3795516

[32] Short A, Wong AK, Finch AM, Haaima G, Shiels IA, Fairlie DP, et al Effects of a new C5a receptor antagonist on C5a- and endotoxin-induced neutropenia in the rat. British Journal of Pharmacology. 1999;126(3):551–4. 10.1038/Sj.Bjp.0702338 WOS:000078693100002. 10188960 PMC1565845

[33] Riedemann NC, Guo RF, Sarma VJ, Laudes IJ, Huber-Lang M, Warner RL, et al Expression and function of the C5a receptor in rat alveolar epithelial cells. Journal of Immunology. 2002;168(4):1919–25. WOS:000173771000054. 10.4049/jimmunol.168.4.1919 11823527

[34] Laudes IJ, Chu JC, Huber-Lang M, Guo RF, Riedemann NC, Sarma JV, et al Expression and function of C5a receptor in mouse microvascular endothelial cells. Journal of Immunology. 2002;169(10):5962–70. WOS:000179170300075. 10.4049/jimmunol.169.10.5962 12421982

[35] Parsons PE, Fowler AA, Hyers TM, Henson PM. Chemotactic Activity in Bronchoalveolar Lavage Fluid from Patients with Adult Respiratory-Distress Syndrome. American Review of Respiratory Disease. 1985;132(3):490–3. WOS:A1985AQL8900005. 4037522 10.1164/arrd.1985.132.3.490

[36] Lomas-Neira J, Chung CS, Perl M, Gregory S, Biffl W, Ayala A. Role of alveolar macrophage and migrating neutrophils in hemorrhage-induced priming for ALI subsequent to septic challenge. American Journal of Physiology-Lung Cellular and Molecular Physiology. 2006;290(1):L51–L8. 10.1152/Ajplung.00028.2005 WOS:000233897700007. 16157517

[37] Lomas-Neira JL, Chung CS, Grutkoski PS, Miller EJ, Ayala A. CXCR2 inhibition suppresses hemorrhage-induced priming for acute lung injury in mice. J Leukoc Biol. 2004;76(1):58–64. 10.1189/jlb.1103541 . 15123771

[38] Wang LY, Han GC, Wang RX, Chen GJ, Xu RN, Xiao H, et al Regulation of IL-8 production by complement-activated product, C5a, in vitro and in vivo during sepsis. Clinical Immunology. 2010;137(1):157–65. 10.1016/J.Clim.2010.05.012 WOS:000282204900018. 20591742

[39] Albertine KH, Soulier MF, Wang Z, Ishizaka A, Hashimoto S, Zimmerman GA, et al Fas and fas ligand are up-regulated in pulmonary edema fluid and lung tissue of patients with acute lung injury and the acute respiratory distress syndrome. Am J Pathol. 2002;161(5):1783–96. 10.1016/S0002-9440(10)64455-0 12414525 PMC1850801

[40] Guo RF, Huber-Lang M, Sarma V, Hlaing T, Shi M, Ward P. Protective effects of anti-C5a in sepsis-induced thymocyte apoptosis. Faseb J. 2001;15(5):A941–A. WOS:000167454201309. 10.1172/JCI10793 PMC381438 11086028

[41] Guo RF, Sun L, Gao H, Shi KX, Rittirsch D, Sarma VJ, et al In vivo regulation of neutrophil apoptosis by C5a during sepsis. J Leukoc Biol. 2006;80(6):1575–83. Epub 2006/09/26. jlb.0106065 [pii] 10.1189/jlb.0106065 . 16997861

[42] Hoth JJ, Wells JD, Brownlee NA, Hiltbold EM, Meredith JW, McCall CE, et al Toll-like receptor 4-dependent responses to lung injury in a murine model of pulmonary contusion. Shock. 2009;31(4):376–81. 18665044 10.1097/SHK.0b013e3181862279 PMC2918369

[43] Hoth JJ, Hudson WP, Brownlee NA, Yoza BK, Hiltbold EM, Meredith JW, et al Toll-like receptor 2 participates in the response to lung injury in a murine model of pulmonary contusion. Shock. 2007;28(4):447–52. . 17558351 10.1097/shk.0b013e318048801a

